# Douleur thoracique aiguë révélant un syndrome de Brugada

**DOI:** 10.11604/pamj.2016.25.95.9442

**Published:** 2016-10-18

**Authors:** Rakotoniaina Masinarivo Daniella, Rasolonjatovo Anjaramalala, Ramiandrisoa, Raveloson Freddie, Rakotoarimanana Solofonirina, Rabearivony Nirina

**Affiliations:** 1 Service de Cardiologie, CHU Joseph Raseta Befelatanana, Antananarivo, Madagascar; 2 Service des Urgences et Soins Intensifs en Cardiologie, CHU Joseph Raseta Befelatanana, Antananarivo, Madagascar

**Keywords:** Douleur thoracique, syndrome de Brugada, mort subite, Chest pain, Brugada syndrome, sudden death

## Abstract

Nous rapportons un cas dans le but d’évaluer le diagnostic électrocardiographique d’un syndrome de Brugada. Il s’agissait d’un homme de 43 ans, d’origine Indienne, qui était venu en consultation pour douleur thoracique aigue. Il était hypertendu grade II non traité et il avait un antécédent de mort subite familial (le père à l’âge de 40 ans). Son ECG montrait un aspect de bloc de branche incomplet droit avec sus décalage descendant du segment ST, avec aspect en dôme au niveau précordial droit et la coronarographie était normale, ce qui nous a permis de poser le diagnostic de syndrome de Brugada de type I. Le patient a pu bénéficier d’une pose de défibrillateur automatique implantable à la Réunion et d’un suivi régulier.

## Abstract (en)

The aim of this case study was to evaluate electrocardiographic diagnosis of Brugada syndrome. Our study involved a 43-year old man of Indian origin coming for consultation due to acute chest pain. The patient suffered from untreated stage 2 hypertension and had a family history of sudden death (his father died at the age of 40 years). ECG showed an incomplete right bundle-branch block with descendant displacement of the ST segment dome-shaped at right precordial level; coronary angiography was normal, which led us to the diagnosis of type I Brugada syndrome. The patient underwent implantation of an implantable automatic defibrillator in Reunion Island and regular follow-up.

## Introduction

Le syndrome de Brugada est une maladie génétique rare dont les manifestations cliniques habituelles sont représentées par syncopes et mort subite survenant chez des sujets jeunes indemnes de toute anomalie cardiaque structurale. Notre objectif est de rapporter un cas de syndrome de Brugada, révélé par une douleur thoracique aigue.

## Patient et observation

**Figure 1:**
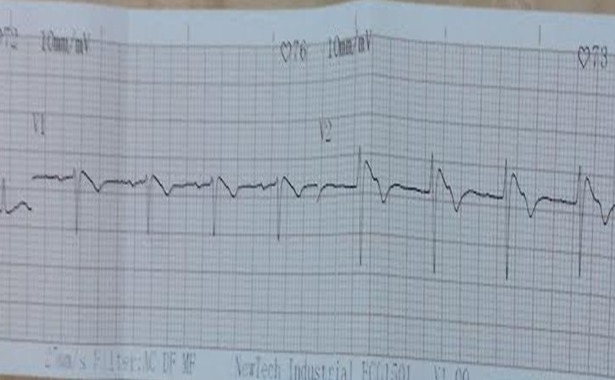
Tracé ECG avec mise en évidence du sus-décalage descendant du segment ST, avec un aspect en dôme ou « coved type » en dérivation précordiale droite (V1-V2) en faveur d’un syndrome de Brugada

Il s’agissait d’un homme, âgé de 43 ans, vu en consultation spécialisée en service de cardiologie pour douleur thoracique. Dans ses antécédents, le patient était hypertendu connu grade II non traité, tabagique à 7PA et il avait des hérédités d’hypertension artérielles et un cas de mort subite non exploré dans la famille celui du père à l’âge de 40 ans. En fait, en novembre 2015, le patient présentait une douleur thoracique médiane, à type de piqûre, d’intensité 5/10 à l’échelle numérique, de durée environ 2 minutes, non irradiée, lors d’un effort de marche, et soulagée par l’arrêt de celui-ci. Les paramètres hémodynamiques étaient corrects et l’examen physique ne révélait rien de particulier. Aux examens paracliniques, le dosage de la troponine était négatif à deux reprises, les autres bilans biologiques étaient normaux. A l’ECG, il y avait un sus décalage de 4 mm du segment ST, en dôme, en précordial droit, sans image en miroir (Figure 1). La coronarographie effectuée n’a objectivé aucune anomalie. Il s’agissait d’un syndrome de Brugada asymptomatique mais avec antécédent familial de mort subite. Le patient a bénéficié d’une pose de défibrillateur automatique implantable à la Réunion et d’une surveillance régulière.

## Discussion

Le syndrome de Brugada est une maladie héréditaire rare mais grave de l’adulte jeune, responsable de syncopes et de morts subites par tachycardie et fibrillation ventriculaire [1]. Il a été découvert pour la première fois en 1992 par les frères Brugada [2]. La prévalence de cette pathologie est de l’ordre de 1 à 5/1000 habitants avec une importante disparité géographique [3]. En effet, le syndrome de Brugada est incriminé comme première cause de décès non traumatique chez les jeunes hommes de moins de 40 ans en Asie du Sud-Est [4]. Il s’agit de la conséquence d’une mutation du gène du canal sodique SNC5A provoquant des perturbations sodiques intra-myocardiques [5]. On peut grouper les patients en deux au cours de cette pathologie: les patients symptomatiques qui se présentent de syncopes ou de lipothymie et les patients asymptomatiques [6]. Dans le cas de notre patient, il a présenté une douleur thoracique à l’effort. Le diagnostic est facile devant un aspect typique à l’ECG qui est un sus-décalage descendant du segment ST supérieur à 0,2 mV sur plus d’une dérivation précordiale droite (V1-V3), avec un aspect en dôme ou « coved type » [3] mis en évidence dans notre cas. En cas de forme frustre ou absence d’anomalie à l’ECG, le diagnostic s’obtient par la réalisation d’un test pharmacologique. Il s’agit d’une injection d’anti-arythmique de classe I (Quinidine, flécainide, procainamide) qui exagère les anomalies du segment ST et démasque le syndrome de Brugada. Sur le plan thérapeutique, en cas de syndrome de Brugada symptomatique, l’implantation d’un défibrillateur automatique implantable reste le traitement de choix [6]. Par contre son indication au cours des formes asymptomatiques reste encore très controversée. Néanmoins, un syndrome de Brugada de type I, l’existence d’un antécédent de mort subite dans la famille du premier degré et l’existence d’une tachycardie ventriculaire soutenue à la stimulation ventriculaire programmée constituent des signes de mauvais pronostic aux cours desquels le DAI est indiqué [1]. Notre patient présente un aspect typique d’un syndrome de Brugada avec mort subite du père à l’âge de 40 ans et il a pu bénéficier d’une implantation de DAI à l’étranger, non encore disponible à Madagascar.

## Conclusion

Le syndrome de Brugada est l’un des diagnostics qu’il faut évoquer en premier lieu chez un patient jeune ayant une syncope. La douleur thoracique ne figure pas parmi les signes cliniques évocateurs. Le diagnostic est facile devant un aspect typique à l’ECG. Actuellement, le défibrillateur automatique implantable reste le seul traitement ayant fait preuve d?efficacité au cours de cette pathologie.
